# Outpatient parenteral antimicrobial therapy (OPAT) for the management of periprosthetic joint infections in the Republic of Ireland (ROI) from 2013 to 2021

**DOI:** 10.1016/j.ijregi.2024.100466

**Published:** 2024-10-11

**Authors:** David Moynan, Paul Reidy, Rhea O'Regan, Fionnuala O'Connor, Eavan G. Muldoon, National OPAT Working Group

**Affiliations:** 1 Department of Infectious Diseases, Mater Misericordiae University Hospital, Dublin, Ireland; 2 Office of the Assistant National Director in Primary Care, Access and Integration, Health Service Executive, Dublin, Ireland

**Keywords:** OPAT, Bone and joint infection, Orthopedic infections, Periprosthetic joint infection, Outpatient parenteral antimicrobial therapy, Service development

## Abstract

**Objectives:**

Periprosthetic joint infection (PJI) is a complication of joint arthroplasty and is seen in 1-2% of cases. Since its initiation in 2013, the national outpatient parenteral antimicrobial therapy (OPAT) program has facilitated the outpatient management of intravenous antimicrobials for PJI. This study aims to describe the clinical epidemiology of patients on OPAT with PJI between 2013 and 2021.

**Methods:**

A retrospective analysis of patients discharged on OPAT between January 1, 2013 to August 31, 2021 was performed using data available from the national OPAT program. This study focused on those with a PJI. Data were analyzed using STATA/SE version 17.0.

**Results:**

From January 1, 2013 until August 31, 2021, there were 14,749 patients managed through the national OPAT program, 8.35% (1232 of 14,749) of which were PJI. Of these, 53% (653 of 1232) were hip arthroplasty, 22.7% (280 of 1232) were knee arthroplasty, and 24.3% (299 of 1232) were “other.” The mean age was 64.5 years (SD 14.15 years). Of those on OPAT, 66.15% (815 of 1232) were health care–administered OPAT, whereas 33.85% (417 of 1232) were self-administered (S) OPAT (S-OPAT). Patients on S-OPAT were statistically younger (61 vs 66 years old, *P* <0.001, 95% confidence interval 14.1-63.6). The most common antimicrobial prescribed was daptomycin (35.8%; 441 of 1232), followed by ceftriaxone (21.2%; 262 of 1232). The median duration on the OPAT program was 27 days (interquartile range 14.5-35 days).

**Conclusion:**

OPAT use in PJI is growing. Cumulatively, it has saved 26,992 hospital bed-days. Although S-OPAT is the preferred strategy and should be considered for all patients, our data demonstrate that health care–administered OPAT is required more frequently in older patients.

## Highlights

• Almost 27,000 hospital bed-days were saved using outpatient parenteral antimicrobial therapy (OPAT) for periprosthetic joint infection management during this study period.
• Self-administered OPAT is more likely to be used in non-Dublin regions.
• Older patients are less likely to be discharged on self-administered OPAT.

## Introduction

The national outpatient parenteral antimicrobial therapy (OPAT) program was established in 2013 as a centralized system that coordinates patients’ OPAT care, accepting referrals from all public hospitals within the Republic of Ireland (ROI). The governance for patient care lies with the local treating infection specialist (infectious diseases physician or clinical microbiologist) [1,2]. It is funded by the Irish Health Service Executive and provided free of charge to all users [1]. It continues to deliver care to patients with complex infections, including orthopedic-related infection [2]. Periprosthetic joint infection (PJI) is a complication of joint arthroplasty that is seen in 1-2% of primary and 4% of revision arthroplasty cases [3]. Between 2013 and 2021, there were 67,353 hip and knee arthroplasties performed in public hospitals and data suggest that demand continues to rise [4,5].

The use of OPAT in orthopedic infections is well-described, although there are limited data on the utility of OPAT in bone and joint infections nationally. The aim of this study is to describe the trends in OPAT practice in relation to PJI between 2013 and 2021, examining not only trends in OPAT delivery (self-administered OPAT [S-OPAT] versus health care–administered OPAT [H-OPAT]) but also antimicrobial use and duration.

## Methods

A retrospective analysis of patients discharged on OPAT between January 1, 2013 to August 31, 2021 was performed using data available from the national OPAT portal, a database to which all patients are enrolled before commencement. Variables including demographics, diagnosis, antimicrobial agent(s), duration of therapy, and method of OPAT delivery were collected. This study focused on PJI, categorized as hip, knee, and “other.” The anonymized data were analyzed using STATA/SE version 17.0. A two-sample *t*-test was used to compare means. Ethical approval was not required for this national service evaluation.

## Results

**Figure 1:**
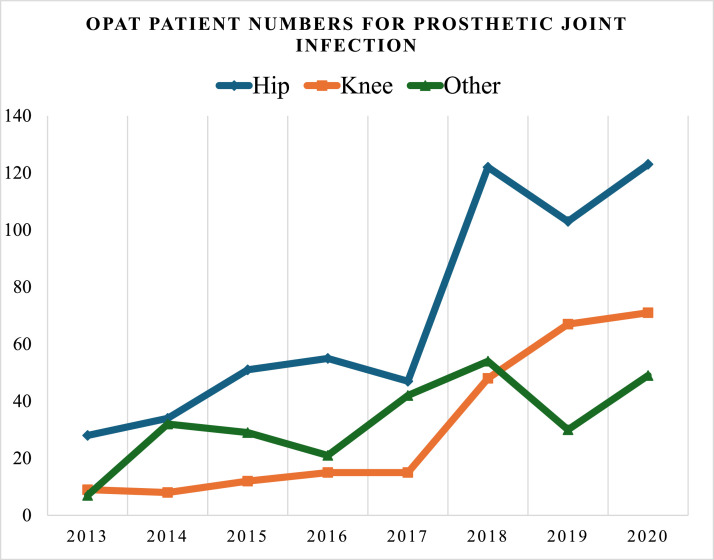
Annual trends in OPAT use for periprosthetic joint infection over the study period. OPAT, outpatient parenteral antimicrobial therapy.

From January 1, 2013 until August 31, 2021, there were 14,749 patients managed through the national OPAT program, 8.35% (1232 of 14,749) of which were PJI. Of these, 53% (653 of 1232) were hip arthroplasty, 22.7% (280 of 1232) were knee arthroplasty, and 24.3% (299 of 1232) were other PJI. Patient numbers have increased each year (Figure 1). The mean age of the cohort was 64.5 years, remaining stable over the study period (65 years in 2013 and 67 years in 2021). There were 13 of 1232 (1%) under the age of 18 years. The majority 66.15% (815 of 1232) were treated with H-OPAT, where the antimicrobial is administered by a health care professional, whereas 33.85% (417 of 1232) were S-OPAT, where the patient or carer administers the antimicrobial. Patients on S-OPAT were statistically younger than those on H-OPAT (61 vs 66 years old, *P* <0.001, 95% confidence interval [CI] 14.1-63.6 years).

The numbers of patients with PJI managed using OPAT varied significantly throughout the country (Table 1). In the ROI, hospitals are defined based on the type of activity provided, ranging from model one to model four. Model one hospitals are community hospitals with no emergency care, whereas model two provides selected acute medicine and a large range of diagnostics. Model three hospitals provide 24/7 acute surgery, acute medicine, and critical care, whereas model four hospitals provide, in addition, tertiary care and often supra-regional care [6]. The top five centers managed over 50% of all PJIs that were discharged on OPAT, with one model four center managing 141 during this study period. The rates of S-OPAT and H-OPAT varied significantly from under 10% utilizing S-OPAT in some centers to over 90% in others. Its use was statistically higher among centers outside of the Dublin region (45% vs 26%; 95% CI 13-24, *P* <0.001).

**Table 1:** OPAT utility in the management of periprosthetic joint infections per center between 2013-2021.

Hospital	Model Hospital	Prosthetic joint infection				S-OPAT N (%)
		Hip	Knee	Other	Total	
A	4	89	24	28	141	23 (16)
B	4	66	32	37	135	50 (37)
C	4	65	36	30	131	33 (25)
D	4	82	18	26	126	10 (8)
E	4	64	31	17	112	41 (37)
F	4	48	20	17	85	75 (88)
G	4	31	15	37	83	38 (47)
H	4	29	28	18	75	34 (45)
I	4	36	9	29	74	18 (24)
J	Other^a^	39	12	23	74	16 (22)
K	3	42	10	10	62	10 (16)
L	3	22	12	8	42	38 (90)
M	3	19	6	2	27	4 (15)
N	3	6	9	3	18	3 (17)
O	2	9	7	0	16	8 (100)
P	3	2	5	3	10	9 (90)
Q	Other^a^	0	0	8	8	2 (25)
R	3	4	0	2	6	2 (33)
S	3	0	6	0	6	3 (50)
T	2	0	0	1	1	0 (0)

OPAT, outpatient parenteral antimicrobial therapy.

a Other; specialist pediatric and orthopedic hospitals.

There was a broad selection of antimicrobials used, most frequently daptomycin (35.8%; 441 of 1232), followed by ceftriaxone (21.2%; 262 of 1232) and flucloxacillin (15/7%; 195 of 1232). Two intravenous antimicrobials were prescribed in 9% (111 of 1232) of patients, increasing over the study period from five cases in 2013 to 22 cases in 2021. The mean duration on the national OPAT program was 26 days (SD 15.2 days, 95% CI 25-27 days). There was a total of 26,992 hospital bed-days saved in patients with a PJI across 20 referring centers.

## Discussion

The national OPAT program continues to grow annually, particularly, in the field of orthopedic-related infections. The data demonstrate a predominant use of H-OPAT, with only one-third of patients self-administering their therapy. S-OPAT numbers have remained stable throughout the study period, accounting for 36% in 2013 and 37% in 2020. The higher proportion of S-OPAT outside of the Dublin region is likely a consequence of the wide geographical distribution across the more rural parts of the country and the limited community nursing availability. However, recent efforts have addressed these geographical variations with community intervention teams now in every county. S-OPAT in the ROI utilizes either an elastomeric device for continuous infusions or pre-compounded antimicrobials for self-administration. Occasionally, antimicrobials requiring reconstitution are prescribed depending on patient capabilities [2]. Unsurprisingly, patients managed with S-OPAT were statistically younger (*P* <0.001) than those on H-OPAT, suggesting a selection bias before recruitment.

Antimicrobial choice is dictated by the offending pathogens in each infection. A significant limitation of this study, however, is the absence of microbiological data in each case because of the database's design. International data demonstrate that *Staphylococcus aureus* and coagulase-negative *Staphylococcus* species account for 50-60% of PJIs, whereas streptococci and enterococci account for roughly 10% of infections [7]. There is, nonetheless, a clear trend toward a once daily antimicrobial or an elastomeric infusion to facilitate administration. There were a significant number of patients in whom two different intravenous antimicrobials were used, increasing over the study period, suggesting increasing complexity.

The national OPAT program plays a pivotal role in the ambulation of patients to the outpatient setting. With respect to PJI, there have been 26,992 bed-days saved since 2013. This translates as a significant cost saving to the health service and enables a better flow of patients within hospitals [2]. The national OPAT guidelines, on behalf of the program, dictates that a weekly review is required in all cases, unless in exceptional circumstances [2]. Outcome data are gathered in each local facility, independent of the national registry analyzed in this study. However, a readmission rate of 34% (12 of 41) was noted in one model four center, with type II diabetes as the biggest risk factor [8]. OPAT clinics are, therefore, a semi-acute facility that continue to monitor the clinical response to treatment and, importantly, ascertain the need for subspecialist input or hospital readmission [2].

There is a significant variation in the volume of PJI managed with OPAT throughout the country, with some centers managing significantly higher numbers than others within the same region. Model four hospitals manage the majority of PJIs on OPAT, likely reflective of the volume of arthroplasty procedures performed and the location of infection specialists. This brings to question whether designated orthopedic-infection centers should be established, chiefly led by orthopedic-infection specialists, because is the case in the United Kingdom, Switzerland, and the United States [9].

## Conclusion

The national OPAT program in the ROI is a comprehensive, nationwide service that has facilitated the management of patients with PJI and continues to grow annually. Although the uptake of S-OPAT has remained around one-third since its inception, patient demographics and physical ability likely influence candidate suitability for this method of delivery. There has been no significant change in the mean age of patients. However, there is an increasing trend of multiple intravenous antimicrobials implemented, suggesting an increasing complexity of patients’ infections.

## Author contributions

All authors provided to the creation of this manuscript. **David Moynan**: conceptualisation, investigation, formal analysis, writing - original draft. **Paul Reidy**: investigation, data curation, resources, writing - review. **Rhea O'Regan**: writing - review. **Fionnuala O'Connor**; data curation, writing - review. **Eavan G Muldoon**: conceptualisation, investigation, writing - review, supervision.

## Declarations of competing interest

The lead author affirms that this manuscript is an honest, accurate, and transparent account of the study being reported and no important aspects have been omitted. The authors have no conflicts of interest to declare and no financial support was provided for the completion of this study.
